# Cardiac rehabilitation may influence leptin and VEGF A crosstalk in patients after acute coronary syndrome

**DOI:** 10.1038/s41598-022-16053-1

**Published:** 2022-07-12

**Authors:** Damian Skrypnik, Katarzyna Skrypnik, Joanna Suliburska, Paweł Bogdański

**Affiliations:** 1 Department of Treatment of Obesity, Metabolic Disorders and Clinical Dietetics, Poznań University of Medical Sciences, Szamarzewskiego St. 82/84, 60-569 Poznan, Poland; 2 Department of Human Nutrition and Dietetics, Poznan University of Life Sciences, Wojska Polskiego St. 31, 60-624 Poznan, Poland

**Keywords:** Physiology, Cardiology, Diseases, Medical research, Risk factors

## Abstract

Leptin, a well-proven cardiovascular risk factor, influences vascular endothelial growth factor A (VEGF A) synthesis via hypoxia-inducible factor 1 alpha (HIF-1A), nuclear factor kappa-light-chain-enhancer of activated B cells (NfkB) and NILCO (Notch, interleukin 1 [IL1] and leptin cross-talk outcome) pathways. This study aimed to investigate the influence of cardiac rehabilitation (CR) on HIF-1A, NfkB and NILCO dependent leptin and VEGF A cross-talk in patients after acute coronary syndrome (ACS). Fifty post-ACS patients underwent a 2-week CR programme (study group S) and were compared to 50 post-ACS subjects who did not undergo CR (control group K). In group S, at baseline and at completion and in group K once, anthropometric, body composition, blood pressure and heart rate measurements were taken and blood sampling was performed. Serum levels of leptin, VEGF A, VEGF receptor 2 (VEGF R2), HIF-1A, NfkB, interleukin 1-alpha (IL1-alpha) and Notch 1 were determined. In group S, serum VEGF A levels increased while leptin, HIF-1A and VEGF R2 levels decreased and completion but not baseline serum leptin correlated positively with serum VEGF A. Also, serum completion VEGF A correlated positively with NfkB and HIF-1A in group S. Correlation analysis in group S confirmed the significant role of the NILCO pathway in the regulation of VEGF A serum levels mediated by HIF-1A and NfkB. CR may induce the predomination of the NILCO pathway interacting with HIF-1A and NfkB over leptin canonical and non-canonical signalling pathways in the leptin influence on VEGF A in post-ACS patients.

**Trial registration:** ClinicalTrials.gov ID: NCT03935438. The CARDIO-REH randomised study.

## Introduction

Ischemic heart disease (IHD) affects approximately 126 million individuals globally, which equates to 1.72% of the world’s population. It is predicted that the prevalence of IHD by 2030 will increase to more than 1845 per 100,000. Currently, IHD leads to approximately nine million deaths annually and has become the leading cause of mortality worldwide^1^. The total 1-year costs of treatment of patient after acute coronary syndrome (ACS) are relatively high, ranging from $34,087 to $86,914 depending on the method of therapy^2^.

Cardiac rehabilitation (CR) with physical exercises is the most significant intervention^3,4^ in patients after ACS and is associated with remarkably more favourable health prognosis compared to patients after ACS who have not undergone CR. The mortality hazard ratio in patients after CR is − 0.47 in comparison to non-rehabilitated patients and the cardiac death rate is reduced (relative risk RR 0.40). CR diminishes the risk of ACS recurrence (RR 0.63) and decreases the frequency of major adverse cardiac events (MACE; RR 0.49)^5^. Thus, it is recommended to implement CR in patients after ACS according to the results of the cardiac stress test (CPX)^4,6^, which has been indicated by the European Society of Cardiology (ESC) as an extensively validated tool in risk stratification in patients with IHD^7^.

Adipose tissue has been recognized as a crucial regulator of cardiovascular health^8^, through the secretion of numerous bioactive molecules, including adipocytokines^9^. Leptin is a cytokine that is mainly produced in adipose tissue^10^. Leptin regulates body mass through food intake inhibition and the stimulation of energy expenditure^11^. In the recent study of Farcas et al. it has been shown, that in patients with coronary artery disease (CAD) and without myocardial infarction (MI) leptin represents a potential mechanism of unfavourable cardiac remodelling: it was associated with left ventricular enddiastolic dimension and left ventricular relative wall thickness^12^. It has been clearly shown that elevated blood leptin levels are associated with the incidence of ACS and predict the short-term occurrence of congestive heart failure (CHF) or cardiac death in patients with CAD independent of obesity status and traditional cardiovascular risk factors^13,14^. Serum leptin concentrations are increased after MI^15^. It is hypothesised that leptin participates in the regulation of cardiac damage development after MI^16^. Interestingly, the results of Nagoya Acute Myocardial Infarction Study shown, that decreased leptin level is connected with a high incidence of adverse events in patients after acute MI^17^. The GG genotype of the − 2548 G/A leptin gene polymorphism is more common in centenarians than in the patients after MI, what indicates that the leptin pathway takes part in the regulation of longevity, presumably by modulating the risk of MI development^18^. All these data show, that the role of leptin in IHD is still not fully clarified and needs investigation. Physical exercise decreases serum leptin levels^19,20^. A recent meta-analysis revealed that the decrease in blood leptin concentrations due to physical training is independent of age and sex and can be observed even after 2 weeks of exercise^21^. Data on the influence of CR on leptin levels after ACS are scant. A limited number of studies have shown that physical training does not influence leptin serum concentrations^22,23^ or prevents blood leptin level increases^24^ in patients with CAD.

Vascular endothelial growth factor (VEGF) is a mitogen that is synthesised by endothelial cells which stimulates angiogenesis and the proliferation of these cells. VEGF is currently reported to be one of the most important proangiogenic factors^25^. VEGF shows mitogenic and chemotactic properties, increases the migration of endothelial cell progenitors and regulates the permeability of blood vessels^26^. With growing serum levels in response to endothelial damage, increased blood concentrations of vascular endothelial growth factor A (VEGFA) have also been stated as an early marker of endothelial dysfunction^27^. A pro-angiogenetic response with an essential role of VEGF A is an effective repair mechanism that has been observed in IHD^28^. Due to endothelial dysfunction, patients with CAD and MI present high serum levels of VEGF A^29^. CR may further increase serum VEGF levels in patients with CAD^30^ and heart failure^31^, possibly reflecting an improvement of endothelial function in response to CR^30^.

In vitro studies have shown that leptin intensifies VEGF A synthesis^32–34^ via the activation of hypoxia-inducible factor 1 alpha (HIF-1A) and nuclear factor kappa-light-chain-enhancer of activated B cells (NfkB)^32^. Moreover, leptin induces the expression and activation of Notch proteins (Notch 1, 2, 3, 4) and up-regulates interleukin 1 (IL1), forming the Notch, IL1 and leptin cross-talk outcome (NILCO). NILCO induces the up-regulation of VEGF and VEGF receptor 2 (VEGF R2)^33^. To date, the role of HIF-1A, NfkB, Notch and IL1 in leptin and VEGF cross-talk has not been investigated in human studies. In our recent study, we showed that the CC genotype of the rs699947 variant of the VEGF gene promotes a positive correlation between leptin and VEGF A blood levels in patients with excess body mass^35^. We also revealed an effect of leptin on VEGF A serum concentration increases in obese women^36^.

The aim of our study was to investigate the influence of a two-week cardiac rehabilitation programme on HIF-1A, NfkB and NILCO-dependent leptin and VEGF A cross-talk in patients after acute coronary syndrome. The novelty of our study is due to the characteristics of the study group, patients after ACS, in which leptin and VEGF A cross-talk has not yet been investigated. In addition, the influence of the studied intervention, cardiac rehabilitation, on leptin and VEGF A interdependency has also not yet been explored.

## Materials and methods

### Study design

The study was designed as a prospective, randomised, interventional and comparative study. The CONSORT guidelines were implemented. The study protocol received the approval of the Bioethics Committee, Poznan University of Medical Sciences (approval no. 476/19) and met the requirements of The Declaration of Helsinki (1975 revision with amendments). The study has been registered on ClinicalTrials.gov under the ID: NCT03935438 (the first registration date 02/05/2019). The study was performed in the Department of Treatment of Obesity, Metabolic Disorders and Clinical Dietetics, Poznan University of Medical Sciences, Poznan, Poland. The study lasted from April 2019 to March 2021.

Subjects fulfilling all inclusion criteria and without any exclusion criteria were enrolled and divided into two groups with an allocation ratio of 1:1: a study group (group S) and a control group (group K). Patients who experienced ACS from 2 to 9 weeks prior to enrolment were included ingroup S. After allocation, patients from group S underwent cardiac rehabilitation lasting for 2 weeks as a study intervention. Patients who experienced ACS within the period of not less than 9 weeks prior to enrolment and had not yet undergone cardiac rehabilitation were included in group K. At baseline and after 2 weeks of intervention, anthropometric and body composition measurements and blood pressure (BP) and heart rate (HR) measurements were taken in patients from group S. Also, blood samples were collected at baseline and at the completion of the study. In patients from group K, the same measurements were taken and blood sampling performed as in patients from group S, but only once. Before the study intervention, the cardiac stress test (CPX) was performed in all patients from group S. According to the recommendations of Polish Cardiac Society (PCS)^4^, a member of the European Society of Cardiology (ESC), CPX was performed in order to determine the patients’ cardiovascular (CV) risk and exercise tolerance and to adjust an effort load during rehabilitation depending on the patients’ health. Patients’ past medical documentation (including data on ACS management and pharmacotherapy, and diabetes) and electrocardiographic (ECG) records within this range were analysed after enrolment by the physician. In all patients, 12-lead electrocardiography was performed directly after enrolment to detect arrhythmias or previously unregistered symptoms of heart ischemia which would constitute a contraindication to physical effort. During the trial, subjects were provided with medical care. There were no important changes to the methods after trial commencement and there were no changes to trial outcomes after the trial commenced. No interim analyses were performed.

### Study patients

Informed consent in writing was obtained from all subjects. Screening and enrolment were performed in the Department of Treatment of Obesity, Metabolic Disorders and Clinical Dietetics, Poznan University of Medical Sciences, Poznan, Poland.

Inclusion criteria were as follows: written informed consent; women and men aged 18 years and more; and ACS in the past: in group S—ACS from 2 to 9 weeks prior to enrolment, in group K—ACS within a period of not less than 9 weeks prior to enrolment without subsequent cardiac rehabilitation. Exclusion criteria were: previous cardiac rehabilitation (phase I cardiac rehabilitation^3^ according to PCS guidelines^4^ in patients from both groups was allowed); a clinically significant acute or chronic inflammatory process in the respiratory, digestive or genitourinary tract or in the mouth, throat or paranasal sinuses, or connective tissue disease; active neoplastic disease; alcohol or drug abuse; pregnancy; lactation; or any other disturbances that in the opinion of the investigators could in any way pose a risk to the patient's health during the study or limit the effectiveness of the study or credibility of results. Also, patients presenting absolute contraindications to exercise testing according to American College of Cardiology/American Heart Association (ACC/AHA) were not included in the study. These contraindications were: high-risk unstable angina, acute myocardial infarction within 2 days, uncontrolled cardiac arrhythmias causing symptoms of hemodynamic compromise, active endocarditis, uncontrolled symptomatic heart failure, symptomatic severe aortic stenosis, acute pulmonary embolus or pulmonary infarction, acute aortic dissection, acute noncardiac disorder that may affect exercise performance or be aggravated by exercise (e.g., infection, renal failure, thyrotoxicosis), acute myocarditis or pericarditis, physical disability that would preclude safe and adequate test performance and an inability to obtain consent^37,38^. Patient sex and age were self-reported. Subjects who met all of the inclusion criteria and did not present any of the exclusion criteria were included in the study. The occurrence of any of exclusion criterion during the trial resulted in withdrawal of the patient from the study.

### Cardiac stress test

In all patients from group S, before the implementation of cardiac rehabilitation, a CPX was performed in order to adjust the effort load during cardiac rehabilitation to the patients’ health state. The basic test was CPX performed according to Bruce's protocol. In patients presenting relative contraindications to exercise testing according to ACC/AHA, instead of Bruce's protocol, a 6 min walk test (6MWT) was performed. These relative contraindications were: moderate stenotic valvular heart disease, left main coronary stenosis or its equivalent, electrolyte abnormalities, tachyarrhythmias or bradyarrhythmia, atrial fibrillation with an uncontrolled ventricular rate, severe arterial hypertension, hypertrophic cardiomyopathy and other forms of outflow tract obstruction, high-degree atrioventricular block and mental impairment leading to an inability to cooperate^37,38^. CPX was performed in the morning, between 8:00 a.m. and 10:00 a.m., under physician supervision in an exercise laboratory. The subjects were instructed not to eat or smoke for 3 h before the CPX and not to perform intense physical effort for at least 12 h before testing^37^.

CPX according to Bruce’s protocol was performed in line with the AHA guidelines^37^. CPX was performed on a treadmill (Aspel B612 model C; Aspel S.A.; Zabierzów; Poland). Before testing, a resting standard 12-lead electrocardiogram (ECG) was obtained and standing BP was measured. During the test, continuous ECG was performed and BP was measured every 3 min. The seven-stage Bruce protocol was used, in which the exercise load increases every 3 min due to an increase in the treadmill velocity and slope angle. In the 1st stage, the treadmill velocity was 1.7 mph (miles per hour) and the %GR (percent grade) was 10; in the 2nd stage, velocity was 2.5 mph and %GR was 12; in the 3rd stage, velocity was 3.4 mph and %GR was 14; in the 4th stage, velocity was 4.2 mph and %GR was 16; in the 5th stage, velocity was 5.0 mph and %GR was 18; in the 6th stage, velocity was 5.5 mph and %GR was 20; and in the 7th stage, velocity was 6.0 mph and %GR was 22. The test was terminated when the pulse limit was reached or when indications to discontinue according to AHA guidelines occurred; these are: ST-segment elevation (> 1.0 mm) in leads without Q waves (other than V1 or aVR); moderate-to-severe angina; drop in systolic blood pressure > 10 mmHg (persistently below baseline); central nervous system symptoms (e.g., ataxia, dizziness, or near syncope); signs of poor perfusion (cyanosis or pallor); sustained ventricular tachycardia; subject’s request to stop; technical difficulties in monitoring the ECG or BP; ST or QRS changes such as excessive ST displacement (horizontal or down-sloping of > 2 mm) or a marked axis shift; increasing chest pain or fatigue, shortness of breath, wheezing, leg cramps, or claudication; hypertensive response (SBP (systolic blood pressure) > 250 mmHg and/or DBP (diastolic blood pressure) > 115 mmHg); arrhythmias other than sustained ventricular tachycardia; and the development of bundle-branch block that cannot be distinguished from ventricular tachycardia^37^. The pulse limit at which CPX was terminated was calculated according to the AHA guidelines as 70% of the age-predicted maximum heart rate. The age-predicted maximum heart rate was calculated by the formula 220—age in years^37^. In the CPX according to Bruce’s protocol: total exercise duration; maximum heart rate (HRmax), maximum BP (BPmax) and metabolic equivalent of task (MET) were measured. A recovery period was included in the observation; in this period, BP was measured and continuous ECG was performed.

In line with the AHA recommendations^37^, in patients from group S presenting relative contraindications to exercise testing according to the ACC/AHA guidelines^37,38^ or with marked left ventricle dysfunction or peripheral arterial occlusive disease who could have not perform CPX according to Bruce’s protocol, the 6MWT was performed^37^. Directly before 6MWT, heart rate, BP and blood oxygen saturation (SO_2_) were measured and a 12-lead ECG was performed. Patients were instructed to walk down a 30-metre corridor at their own pace and attempt to cover as much ground as possible in 6 min. At the end of the 6-min period, the patients discontinued walking, the total distance walked was determined and the symptoms reported by the subject were recorded. Directly after walk termination, maximum heart rate (HRmax); maximum BP (BPmax) and SO_2_ were measured. HR, BP and SO_2_ were also measured 1 min and 2 min after the walk terminated^37^. Mean walk velocity was determined and MET was calculated according to the formula MET = [0.1 × velocity (m∙min^−1^) + 3.5mLO_2_∙kg∙min^−1^] ÷ 3.5mLO_2_ kg min^−1^^39^. After termination of the 6MWT, the 12-lead ECG was repeated.

### Anthropometric parameters and body composition measurement

Body composition analyses and anthropometric measurements were taken in the metabolic laboratory in patients wearing no shoes and light clothes, in the morning, after a night-long sleep, and with the patient fasting. Height was measured to the nearest 0.1 cm with the use of manual stadiometer and body mass was measured to the nearest 0.1 kg with the use of electronic scales (InBody 370 device, InBody Bldg, Seoul, Korea). Body mass index (BMI) was calculated as the body mass divided by the height squared (kg/m^2^). The hip circumference (HC) was measured at the maximum protuberance of the buttocks. The waist circumference (WC) was measured at the end of normal expiration in the horizontal plane midway between the lowest rib and the iliac crest. WC and HC were measured with the use of non-stretchable plastic tape to the nearest 0.5 cm in standing position.

Body composition measurements were performed with the use of the electrical bioimpedance method using the InBody 370 device (InBody Bldg, Seoul, Korea). The body composition analysis procedure lasted for about 60–90 s. Before the measurements, the patients cleaned their hands and feet using antibacterial solution. Data of subjects, including sex, body height and age, were recorded on the device. Subsequently, the patients assumed an upright motionless position on the device with their feet centred on the lower electrodes and with their hands grabbing the upper electrodes. Arms were positioned wide to avoid contact with the torso. After the procedure, the subjects were asked to step down from the device. Percentage fat tissue content (%FTC), mass fat tissue content (FTC), fat-free mass (FFM),muscle mass (MM) and basal metabolic rate (BMR) were registered.

### Blood pressure measurements

Blood pressure measurements were performed with the use of a digital electronic tensiometer (model 705IT TM, Omron Corporation, Kyoto, Japan). During the measurement procedure, subjects were sitting in a chair for > 5 min with a supported back and with their feet on the floor, after relaxation, with an empty bladder. Caffeine consumption and physical exertion for at least 30 min before measurement was forbidden. During the measurements, the subject’s arm was supported resting on a table. Standard or large-size cuffs for adults were used. BP was measured in both the left and right arms; the arm for which higher BP value was read was used for subsequent measurements. Three further measurements were taken and the mean was calculated and recorded. HR was determined under the same conditions by stethoscopic auscultation of the heart.

### Blood sample collection and biochemical analysis

Blood samples were collected in the morning, after a full-night sleep, at room temperature, with the patient fasting and without prior caffeine intake. Before collection, the subjects lay supine for 30 min in silence. Blood samples were collected into serum separated tubes from the ulnar vein. After preparation, serum samples were stored at − 80 °C.

Serum concentrations of leptin, VEGF A, VEGF R2, HIF-1A, NfkB, interleukin 1-alpha (IL1-alpha) and Notch 1 have been determined with enzyme-linked immunosorbent assay (ELISA) and commercial kits (leptin, VEGF A, Notch 1—Ray Biotech, Peachtree Corners, Georgia, USA; VEGF R2, NfkB, IL1-alpha—ELK Biotechnology, Wuhan, China; HIF-1A –Elabscience, Houston, Texas, USA). Spectrometry was performed with Infinite F50 spectrometer (Tecan Group Ltd., Männedorf, Switzerland).

### Intervention

Patients from group S underwent phase II of cardiac rehabilitation^3^ according to PCS guidelines^4^ in a cardiac rehabilitation medical centre. The intervention lasted for 2 weeks. The cardiac rehabilitation programme was conducted by a trained physiotherapist under medical supervision in the cardiac rehabilitation room. Directly before, after and in the middle of the exercise set, the patient’s BP and HR were measured and recorded. In order to increase patient safety, an ECG monitor, cardiac defibrillator, resuscitation equipment and medication were directly available and a resuscitation team consisting of anaesthesiologist and medical rescuer was immediately contactable.

The patients were qualified for the cardiac rehabilitation programme by a physician based on the results of the CPX and the patients’ medical history. The programme was adjusted to the patients’ health state and exercise tolerance according to PCS recommendations^4^. The cardiac rehabilitation programme consisted of free active exercises, isometric exercises, isotonic exercises, isokinetic exercises, active resistance exercises, active breathing exercises, active breathing exercises with resistance, balance exercises, individual and group general fitness exercises, interval and continuous training on a bicycle cycloergometer (Aspel CRG 200; Aspel, Zabierzów, Poland) under continuous ECG monitoring (Aster Beta System XL; Aspel, Zabierzów, Poland), station training, marching training, marching training with equipment and walking. Cardiac rehabilitation took the form of individual training or training in small groups consisting of 2–4 patients.

The training took place on each day of the rehabilitation programme, for 14 days in total, without any break. There were three training sessions per day, with a total daily duration of 30–90 min depending on the patients’ health and exercise tolerance according to PCS recommendations^4^. The sessions started everyday at 8:00 a.m., 10:30 a.m. and 01:00 p.m. Each session began with a 5–10 min aerobic warm-up followed by the main cardiac rehabilitation training. The session was completed with a 5–10 min session of breathing and stretching, with cool-down aerobic exercises.

Due to the PCS recommendations^4^, the main cardiac rehabilitation training was performed in accordance with one of four models (A, B, C or D), depending on the patients’ cardiovascular risk grade and performance on CPX. Patients were allocated to model A, B, C or D by a physician. The main models of cardiac rehabilitation training according to PCS recommendations are presented in Table 1^4^. Heart rate reserve (HRR) was calculated according to PCS recommendations with the formula: HRR = maximum heart rate during CPX (HRmax) - resting heart rate (HRrest)^4^. The patients’ cardiovascular risk grade stratification according to PCS guidelines are presented in Table 2^4^. Except for the cardiac rehabilitation training implemented in group S, there were no differences between groups S and K in investigation procedures.

**Table 1:** Models of main cardiac rehabilitation training according to Polish Cardiac Society guidelines^4^.

Model	CV risk	CPX result–exercise tolerance	Total daily training duration [min]	Training intensity
A	Low	≥ 7 MET	60–90	60–80% HRR or 50–70% HRmax
B	Mid	≥ 5 MET	45–60	50–60% HRR or 50% HRmax
C	Mid	3–5 MET	45	40–50% HRR or 40–50% HRmax
	High	≥ 6 MET		
D	Mid	< 3 MET	30–45	< 20% HRR or < 110–115% HRrest
	High	< 6 MET		

CPX: cardiac stress test; CV: cardiovascular; HRmax: maximum heart rate during CPX; HRrest: resting heart rate; HRR: heart rate reserve; MET: metabolic equivalent of task; min: minutes; HRR = maximum heart rate during CPX – resting heart rate^4^.

**Table 2:** Patient’s cardiovascular risk grade stratification according to Polish Cardiac Society guidelines^4^.

Parameter	Cardiovascular risk grade		
	Low^a^	Mid^b^	High^b^
LVEF	≥ 50%	36–49%	≤ 35%
Complex ventricular arrhythmia	Absent at rest and during exercise		Present at rest and during exercise
Symptoms of ischemia on an exercise ECG	Absent	ST segment depression ≥ 1 mm and ≤ 2 mm	ST segment depression > 2 mm
Exercise tolerance based on CPX	≥ 7 MET	5–6.9 MET	< 5 MET
Hemodynamic reaction to exercise during CPX	normal		No increase or decrease in SBP or HR with increasing exercise load
Clinical data	Uncomplicated MI, CABG, PCI		MI or cardiac intervention (e.g. PCI, CABG) complicated by shock; heart failure; recurrences of ischemia after invasive treatment

CABG: coronary artery bypass grafting; CPX: cardiac stress test; ECG: electrocardiogram; HR: heart rate; LVEF: left ventricular ejection fraction (the most current data based on medical documentation was used); MET: metabolic equivalent of task; MI: myocardial infarction; PCI: percutaneous coronary intervention; SBP: systolic blood pressure.

^a^All criteria present; ^b^at least one criterion present.

### Patient allocation and statistical analysis

All subjects included in the study were assigned a unique code as an identifier by one person from the study team. Throughout the whole study, only this person had access to the database enabling the identification of patients’ personal data with the use of codes. Subsequently, this study team member prepared the randomisation list of codes without patients’ data. Afterwards, patients were randomly allocated to group S or K in allocation ratio 1:1 using the randomisation list by a physician, who assigned participants to the intervention or control group, respectively. The random allocation sequence was computer-generated. The only restriction of randomization were inclusion and exclusion criteria: in group S this was ACS from 2 to 9 weeks prior to enrolment, while in group K it was ACS within a period of no less than 9 weeks prior to enrolment. The study personnel, except for the individual who assigned a unique code to subjects, was blinded throughout the whole study. The person assigning a unique code to subjects did not participate in any other study procedures, including data assessment and analysis.

Statistical analysis of data was done using TIBCO Software Inc. (2017), Statistica (data analysis software system), version 13. All results were first verified by a normality test. Since the test confirmed a lack of normality, non-parametric methods were used for statistical analysis. The difference between the results before and after rehabilitation was checked using the Wilcoxon test while the difference between the groups was checked using the Mann–Whitney test. The relationship between the selected variables was assessed using the Spearman rank correlation test. Additionally, the relationship between serum level of leptin (x) and VEGF A (y) was checked using a linear regression model (y = β_1_x + β_0_). Patients’ sex distribution between study groups was compared using Fisher’s test. Results were accepted as significant at p < 0.05.

Due to the R-values, correlations were considered as weak (R 0.0–0.3); moderate (R 0.3–0.5); strong (R 0.5–0.7) or very strong (R > 0.7)^40^. No additional analyses, such as adjusted analyses, were performed.

## Results

**Figure 1:**
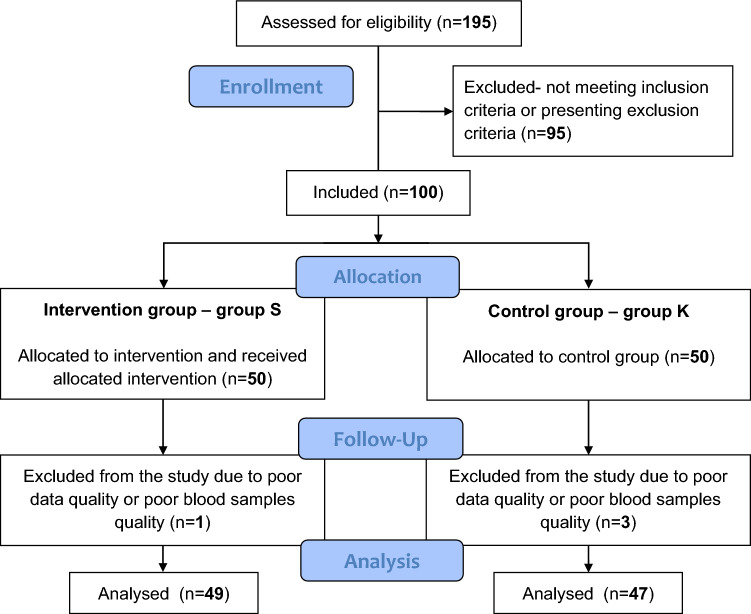
The study flow diagram.

A random cohort of 195 patients from the Department of Cardiac Rehabilitation and the Department of Hypertension and Metabolic Disorders; Poznan University Hospital of the Lord’s Transfiguration, Poznan, Poland, who had experienced ACS, was screened. Ninety-five patients were not included as they did not meet the inclusion criteria or presented exclusion criteria. A total of 100 subjects were enrolled and allocated into group S (n = 50) or group K (n = 50). All patients from group S and K underwent data collection, measurement procedures and blood sample collection, with all patients from group S also receiving the study intervention. One participant from group S and three participants from group K were excluded from the study after the completion of data collection, measurement procedures, blood sample collection and study intervention due to poor quality data or poor blood sample quality. A total of 96 patients underwent statistical analysis, 49 from group S and 47 from group K. The study flow diagram is presented in Fig. 1. The trial was completed when the intervention of the last patient from group S had ended and data collection, measurement procedures and blood sample collection from all patients from groups S and K were completed. There were no important harms or unintended effects in the study groups. The analyses were performed by the original assigned groups. No ancillary analyses were performed.

The baseline characteristics of groups S and K are presented in Table 3. At baseline there was no significant difference in patient age between the study groups (p-value = 0.6095). Also, there was no significant difference in patient sex distribution between the study groups (group S: women 17, men 32; group K: women 13, men 34; Fisher’s p-value = 0.5132). In the whole study population 38 patients had diabetes. At enrolment median post-ACS time was 3 weeks in group S and 29 weeks in group K (Mann–Whitney test p-value: < 0.0001). In group S at baseline, before the study intervention, CPX was performed to adjust the effort load during cardiac rehabilitation according to the patients’ health state. In group S, 10 patients underwent CPX according to Bruce's protocol and 39 patients who presented relative contraindications to exercise testing according to ACC/AHA guidelines^37,38^ underwent the 6MWT instead. The baseline characteristics of CPX performed in group S are presented in Table 4.

**Table 3:** Baseline characteristics of group S and K.

Parameter	Group S	Group K	p-value
n	49	47	
Age [years]	65 [59; 69]	65 [58; 73]	0.6095^#^
Height [cm]	168.5 [163.0; 177.0]	168.0 [161.5; 174.0]	0.4549^#^
Body mass [kg]	77.4 [67.5; 85.0]	84.7 [79.8; 102.3]	0.0045^#^
BMI [kg/m^2^]	26.4 [23.8; 30.3]	30.0 [26.0; 34.5]	0.0011^#^
Leptin [ng/ml]	1.68 [1.10; 2.90]	3.20 [1.57; 5.57]	0.0014^#^
VEGF A [pg/ml]	72.55 [50.59; 147.03]	95.42 [63.87; 162.97]	0.1528^#^
SBP [mmHg]	120.0 [111.0; 135.0]	140.5 [128.0; 154.0]	0.0002^#^
DBP [mmHg]	73.0 [66.0; 83.0]	78 [68.5; 91.0]	0.2621^#^
HR [bpm]	66.0 [61.0; 75.0]	66.5 [60.0; 74.0]	0.7647^#^

Data are presented as median [Q1; Q3]. BMI: body mass index; bpm: beats per minute; DBP: diastolic blood pressure; HR: heart rate; Q1: first quartile; Q3: third quartile; SBP: systolic blood pressure; VEGF A: vascular endothelial growth factor A.

^#^Mann–Whitney test.

**Table 4:** Baseline characteristics of group S–CPX results.

Bruce's protocol		6 min walk test	
Parameter	Median [Q1; Q3]	Parameter	Median [Q1; Q3]
n	10	n	39
Total exercise duration [s]	393 [361; 563]	HRmax [bpm]	78 [71; 82]
HRmax [bpm]	135 [125; 136]	SBPmax [mmHg]	132 [112; 148]
SBPmax [mmHg]	140 [130; 160]	DBPmax [mmHg]	71 [60; 79]
DBPmax [mmHg]	80 [70; 90]	SO_2_ [%]	98 [97; 99]
Metabolic equivalent of task [MET]	7.4 [3.7; 10.4]	Total distance walked [m]	390 [300; 480]
		Walk velocity [m/min]	65 [50; 80]
		Metabolic equivalent of task [MET]	2.86 [2.43; 3.29]

Data are presented as median [Q1; Q3]. bpm: beats per minute; CPX: cardiac stress test; DBPmax: maximum diastolic blood pressure; HRmax: maximum heart rate; MET: metabolic equivalent of task; Q1: first quartile; Q3: third quartile; SBPmax: maximum systolic blood pressure; SO_2_: blood oxygen saturation at the completion of 6 min walk test.

In the whole study population ACS was managed with the use of: percutaneous coronary intervention (PCI) with drug eluting stent (DES) implantation: 87 patients; PCI and thrombectomy: 1 patient; coronary artery bypass grafting (CABG): 2; plain old balloon angioplasty (POBA): 2 patients; conservative treatment strategy: 4 patients.

In group S the following pharmacotherapy was used: dual antiplatelet therapy (DAPT): 46 patients; single antiplatelet therapy (SAPT): 2 patients; no antiplatelet therapy: 1 patient; beta-blocker: 48 patients; angiotensin-converting-enzyme inhibitors (ACEI): 37 patients; angiotensin receptor blockers (ARB): 9 patients; HMG-CoA (3-hydroxy-3-methylglutaryl coenzyme A) reductase inhibitors (statins): 47 patients.

In group K the following pharmacotherapy was used: DAPT: 17 patients; SAPT: 28 patients; no antiplatelet therapy: 2 patients; beta-blocker: 42 patients; ACEI: 29 patients; ARB: 10 patients; HMG-CoA reductase inhibitors (statins): 41 patients.

### Anthropometric and body composition

In group S, the patients’ body mass and BMI after the intervention were higher compared to baseline. Body mass and BMI in group S were lower compared to group K, both at baseline and at completion. HC and WC were higher in group K compared to group S before and after the intervention. There were no differences in %FTC between the groups; at baseline, FTC was lower in group S compared to group K, however, the significance of this difference is borderline. In group S, FFM, MM and BMR were lower after the intervention compared to those at study onset. Anthropometric and body composition results are shown in Table 5.

**Table 5:** Anthropometric, body composition analysis, resting blood pressure and resting heart rate results.

Parameter	Group	Median [Q1; Q3]	p-value	
Body height [cm]	S (I)	168.5 [163.0; 177.0]	S (I) vs. K	0.4549^#^
	K	168.0 [161.50; 174.00]		
Body mass [kg]	S (I)	77.4 [67.5; 85.0]	S (I) vs. S (II)	0.0294*
	S (II)	77.7 [66.6; 84.5]	S (I) vs. K	**0.0045**^#^
	K	84.7 [79.8; 102.3]	S (II) vs. K	**0.0032**^#^
BMI [kg/m^2^]	S (I)	26.4 [23.8; 30.3]	S (I) vs. S (II)	0.0107*
	S (II)	26.9 [23.2; 30.7]	S (I) vs. K	**0.0011**^#^
	K	30.0 [26.0; 34.5]	S (II) vs. K	** < 0.0001**^#^
HC [cm]	S (I)	102.0 [96.5; 110.0]	S (I) vs. S (II)	0.6402*
	S (II)	100.0 [97.0; 109.0]	S (I) vs. K	**0.0015**^#^
	K	109.0 [102; 118.5]	S (II) vs. K	**0.0006**^#^
WC [cm]	S (I)	97.0 [92.0; 107.0]	S (I) vs. S (II)	0.4001*
	S (II)	98.5 [91.5; 106.0]	S (I) vs. K	**0.0029**^#^
	K	106.0 [98.0; 117.0]	S (II) vs. K	**0.0060**^#^
%FTC [%]	S (I)	31.4 [25.1; 41.4]	S (I) vs. S (II)	0.0804*
	S (II)	31.6 [25.5; 40.4]	S (I) vs. K	0.0863^#^
	K	35.3 [31.4; 43.6]	S (II) vs. K	0.0996^#^
FTC [kg]	S (I)	22.6 [19.3; 31.4]	S (I) vs. S (II)	0.4506*
	S (II)	23.4 [19.7; 31.5]	S (I) vs. K	**0.0445**^#^
	K	29.8 [21.3; 42.5]	S (II) vs. K	0.0568^#^
FFM [kg]	S (I)	53.8 [45.4; 61.2]	S (I) vs. S (II)	0.0139*
	S (II)	52.9 [42.8; 58.1]	S (I) vs. K	0.6761^#^
	K	52.8 ± [47.3; 60.9]	S (II) vs. K	0.4364^#^
MM [kg]	S (I)	29.9 [24.6; 33.7]	S (I) vs. S (II)	0.0052*
	S (II)	29.3 [23.0; 32.6]	S (I) vs. K	0.5121^#^
	K	29.6 [25.6; 34.4]	S (II) vs. K	0.2882^#^
BMR [kcal]	S (I)	1532.0 [1350.0; 1690.5]	S (I) vs. S (II)	0.0101*
	S (II)	1512.0 [1294.0; 1625.0]	S (I) vs. K	0.6761^#^
	K	1510.0 [1392.0; 1685.0]	S (II) vs. K	0.4364^#^
SBP [mmHg]	S (I)	120.0 [111.0; 135.0]	S (I) vs. S (II)	0.0028*
	S (II)	114.5 [100.0; 130.0]	S (I) vs. K	**0.0002**^#^
	K	140.5 [128.0; 154.0]	S (II) vs. K	** < 0.0001**^#^
DBP [mmHg]	S (I)	73.0 [66.0; 83.0]	S (I) vs. S (II)	0.0060*
	S (II)	68.5 [62.0; 78.0]	S (I) vs. K	0.2621^#^
	K	78.0 [68.5; 91.0]	S (II) vs. K	**0.0013**^#^
HR [bpm]	S (I)	66.0 [61.0; 75.0]	S (I) vs. S (II)	0.5127*
	S (II)	65.0 [59.0; 72.0]	S (I) vs. K	0.7647^#^
	K	66.5 [60.0; 74.0]	S (II) vs. K	0.3216^#^

Data are presented as median [Q1; Q3]. %FTC: percentage fat tissue content; BMI: body mass index; BMR: basal metabolic rate; bpm: beats per minute; DBP: resting diastolic blood pressure; FFM: fat-free mass; FTC: mass fat tissue content; HC: hip circumference; HR: resting heart rate; MM: muscle mass; Q1: first quartile; Q3: third quartile; SBP: resting systolic blood pressure; WC: waist circumference.

(I): value before intervention; (II): value after the intervention.

*Wilcoxon test; ^#^Mann–Whitney test.

Significant values are given in bold.

### Blood pressure and heart rate

In group S, both SBP and DBP were lower after the intervention compared to at baseline. When comparing group S to group K, it was seen that blood pressure was higher in the second group in the case of SBP at both baseline and completion and in the case of DBP at the end of the intervention only. There were no differences in patient HR in group S between study onset and study completion, or between groups S and K. Blood pressure and heart rate results are shown in Table 5.

### Biochemical results

In group S, serum VEGF A level was higher and serum leptin level was lower after the intervention compared to baseline. Also, leptin serum concentrations were higher in group K compared to group S, both at study onset and at study completion. In group S, HIF-1A and VEGF R2 serum levels were lower after the intervention compared to those at baseline. HIF-1A and NfkB serum concentrations were lower in group S after the intervention compared to group K. There were no differences in serum levels of Notch 1 and IL1-alpha, neither in group S between study onset and completion, nor between groups S and K. The results of serum biochemical analyses are presented in Table 6.

**Table 6:** Serum concentration of biochemical parameters.

Parameter	Group	Median [Q1; Q3]	p-value	
VEGF A [pg/ml]	S (I)	72.55 [50.59; 147.03]	S (I) vs. S (II)	0.0003*
	S (II)	123.75 [81.48; 169.49]	S (I) vs. K	0.1528^#^
	K	95.42 [63.87; 162.97]	S (II) vs. K	0.1505^#^
Leptin [ng/ml]	S (I)	1.68 [1.10; 2.90]	S (I) vs. S (II)	0.0100*
	S (II)	1.23 [0.46; 2.80]	S (I) vs. K	**0.0014**^#^
	K	3.20 [1.57; 5.57]	S (II) vs. K	**0.0001**^#^
HIF-1A [pg/ml]	S (I)	61.61 [28.24; 107.74]	S (I) vs. S (II)	0.0167*
	S (II)	24.14 [13.06; 105.95]	S (I) vs. K	0.2816^#^
	K	93.95 [34.06; 332.09]	S (II) vs. K	**0.0047**^#^
NfkB [ng/ml]	S (I)	0.35 [0.28; 0.48]	S (I) vs. S (II)	0.0808*
	S (II)	0.26 [0.22; 0.44]	S (I) vs. K	0.2247^#^
	K	0.39 [0.28; 0.62]	S (II) vs. K	**0.0011**^#^
Notch 1 [pg/ml]	S (I)	718.53 [643.14; 937.36]	S (I) vs. S (II)	0.4233*
	S (II)	738.12 [620.22; 1085.20]	S (I) vs. K	0.5756^#^
	K	899.74 [593.29; 1413.70]	S (II) vs. K	0.8039^#^
IL1-alpha [pg/ml]	S (I)	32.87 [22.89; 47.15]	S (I) vs. S (II)	0.9564*
	S (II)	32.87 [24.22; 47.15]	S (I) vs. K	0.2546^#^
	K	26.30 [22.31; 52.83]	S (II) vs. K	0.2579^#^
VEGF R2 [ng/ml]	S (I)	0.21 [0.14; 0.29]	S (I) vs. S (II)	0.0229*
	S (II)	0.17 [0.14; 0.21]	S (I) vs. K	0.3905^#^
	K	0.19 [0.15; 0.23]	S (II) vs. K	0.1680^#^

Data are presented as median [Q1; Q3]. HIF-1A, hypoxia-inducible factor 1 alpha; IL1-alpha, interleukin 1 alpha; NfkB, nuclear factor kappa-light-chain-enhancer of activated B cells; Notch 1, Notch protein 1; Q1: first quartile; Q3: third quartile; VEGF A, vascular endothelial growth factor A; VEGF R2, vascular endothelial growth factor receptor 2.

(I): value before intervention; (II): value after the intervention.

*Wilcoxon test; ^#^Mann–Whitney test.

Significant values are given in bold.

In group S, it has been shown that leptin serum concentrations correlated positively with serum VEGF A level after the intervention. No such correlation was found for baseline leptin or group K. In group S, serum VEGF A concentration correlated positively with NfkB and HIF-1A blood levels after the intervention. These correlations seem to be one of the key findings of the study. Correlations between biochemical parameters and between leptin and VEGF A and anthropometric, body composition parameters, BP and HR in group S are shown in Table 7.

**Table 7:** Significant correlations between registered parameters in group S.

**Correlations between biochemical parameters**					
Leptin (II) & VEGF A (I)	0.29 [0.0418]	Δ VEGF A & HIF-1A (I)	0.37 [0.0083]	NfkB (I) & VEGF R2 (I)	0.32 [0.0332]
Leptin (II) & VEGF A (II)	0.30 [0.0366]	Δ VEGF A & IL1-alpha (I)	0.33 [0.0187]	NfkB (I) & Δ VEGF R2	− 0.31 [0.0391]
Leptin (I) & Notch 1 (II)	0.33 [0.0211]	HIF-1A (I) & NfkB (I)	0.42 [0.0031]	NfkB (II) & Notch 1 (I)	0.29 [0.0434]
Δ Leptin & Notch 1 (II)	− 0.35 [0.0138]	HIF-1A (I) & IL1-alpha (I)	0.49 [0.0004]	NfkB (II) & Notch 1 (II)	0.41 [0.0421]
VEGF A (I) & HIF-1A (II)	0.32 [0.0249]	HIF-1A (I) & Notch 1 (I)	0.32 [0.0266]	NfkB (I) & IL1-alpha (I)	0.55 [0.0001]
VEGF A (II) & NfkB (I)	0.42 [0.0028]	HIF-1A (I) & Notch 1 (II)	0.30 [0.0364]	Δ NfkB & IL1-alpha (I)	− 0.35 [0.0134]
VEGF A (II) & NfkB (II)	0.30 [0.0351]	HIF-1A (II) & IL1-alpha (II)	0.38 [0.0066]	Δ NfkB & VEGF R2 (I)	− 0.39 [0.0090]
VEGF A (II) & HIF-1A (I)	0.29 [0.0467]	Δ HIF-1A & IL1-alpha (I)	− 0.41 [0.0033]	Δ NfkB & Notch 1 (II)	0.30 [0.0404]
VEGF A (II) & HIF-1A (II)	0.39 [0.0057]	Δ HIF-1A & Notch 1 (I)	− 0.32 [0.0242]	VEGF R2 (I) & IL1-alpha (I)	0.40 [0.0070]
**Correlations between leptin and anthropometric, body composition parameters, BP and HR**					
Leptin (I) & Δ body mass	− 0.38 [0.0123]	Leptin (I) & FTC (II)	0.78 [< 0.0001]	Leptin (II) & %FTC (II)	0.74 [< 0.0001]
Leptin (I) & BMI (I)	0.57 [0.0001]	Leptin (I) & Δ FTC	− 0.46 [0.0082]	Leptin (II) & Δ %FTC	− 0.35 [0.0492]
Leptin (I) & BMI (II)	0.53 [0.0003]	Leptin (II) & BMI (I)	0.48 [0.0005]	Leptin (II) & BMR (I)	− 0.34 [0.0405]
Leptin (I) & Δ BMI	− 0.47 [0.0044]	Leptin (II) & BMI (II)	0.43 [0.0042]	Leptin (II) & MM (I)	− 0.35 [0.0352]
Leptin (I) & WC (I)	0.44 [0.0023]	Leptin (II) & Δ BMI	− 0.36 [0.0198]	Leptin (II) & FTC (I)	0.61 [< 0.0001]
Leptin (I) & WC (II)	0.52 [0.0006]	Leptin (II) & WC (I)	0.31 [0.0415]	Leptin (II) & FTC (II)	0.67 [< 0.0001]
Leptin (I) & HC (I)	0.45 [0.0022]	Leptin (II) & WC (II)	0.42 [0.0075]	Leptin (II) & Δ FTC	− 0.52 [0.0492]
Leptin (I) & HC (II)	0.49 [0.0016]	Leptin (II) & HC (I)	0.39 [0.0089]	Leptin (II) & FFM (I)	− 0.34 [0.0405]
Leptin (I) & SBP (II)	0.35 [0.0019]	Leptin (II) & HC (II)	0.42 [0.0082]	Δ Leptin & SBP (I)	0.39 [0.0013]
Leptin (I) & %FTC (I)	0.75 [< 0.0001]	Leptin (II) & SBP (I)	0.37 [0.0087]	Δ Leptin & DBP (I)	0.37 [0.0424]
Leptin (I) & %FTC (II)	0.79 [< 0.0001]	Leptin (II) & SBP (II)	0.34 [0.0192]	Δ Leptin & Δ SBP	− 0.29 [0.0497]
Leptin (I) & FTC (I)	0.76 [< 0.0001]	Leptin (II) & %FTC (I)	0.71 [< 0.0001]	Δ Leptin & Δ DBP	− 0.42 [0.0037]
**Correlations between VEGF A and anthropometric, body composition parameters, BP and HR**					
VEGF A (I) & HR (I)	0.32 [0.0236]	VEGF A (I) & Δ FTC	− 0.45 [0.0098]	VEGF A (II) & Δ FTC	− 0.37 [0.0345]
VEGF A (I) & Δ SBP	− 0.30 [0.0414]	VEGF A (I) & Δ FFM	0.35 [0.0483]	Δ VEGF A & FTC (I)	− 0.38 [0.0481]
VEGF A (I) & FTC (I)	0.34 [0.0447]				

Data presented as the Spearman correlation coefficient R value; p value is presented below R value in square bracket []. %FTC: percentage fat tissue content; BMI: body mass index; BMR: basal metabolic rate; DBP: resting diastolic blood pressure; FFM: fat-free mass; FTC: mass fat tissue content; HC: hip circumference; HIF-1A: hypoxia-inducible factor 1 alpha; HR: resting heart rate; IL1-alpha: interleukin 1 alpha; MM: muscle mass; NfkB: nuclear factor kappa-light-chain-enhancer of activated B cells; Notch 1: Notch protein 1; SBP: resting systolic blood pressure; VEGF A: vascular endothelial growth factor A; VEGF R2: vascular endothelial growth factor receptor 2; WC: waist circumference.

(I): value before intervention; (II): value after the intervention; Δ: change/delta = value in group S (II) – value in group S (I).

In group K, a positive correlation between serum HIF-1A and NfkB concentrations was found. In this group, no correlation between serum levels of leptin and VEGF A was shown. Correlations between biochemical parameters and between leptin and VEGF A and anthropometric, body composition parameters, BP and HR in group K are shown in Table 8.

**Table 8:** Significant correlations between registered parameters in group K.

**Correlations between biochemical parameters**					
HIF-1A & IL1-alpha	0.51 [0.0005]	HIF-1A & Notch 1	0.37 [0.0160]	IL1-alpha & NfkB	0.62 [< 0.0001]
HIF-1A & NfkB	0.65 [< 0.0001]	IL1-alpha & VEGF A	0.34 [0.0242]		
**Correlations between leptin and VEGF A and anthropometric, body composition parameters, BP and HR**					
Leptin & WC	0.44 [0.0064]	Leptin & SBP	0.39 [0.0218]	Leptin & FTC	0.71 [0.0002]
Leptin & HC	0.41 [0.0126]	Leptin & %FTC	0.87 [< 0.0001]	VEGF A & DBP	− 0.43 [0.0137]

Data presented as the Spearman correlation coefficient R value; p value is presented below R value in square bracket []. %FTC: percentage fat tissue content; DBP: resting diastolic blood pressure; FTC: mass fat tissue content; HC: hip circumference; HIF-1A: hypoxia-inducible factor 1 alpha; IL1-alpha: interleukin 1 alpha; NfkB: nuclear factor kappa-light-chain-enhancer of activated B cells; Notch 1: Notch protein 1; SBP: resting systolic blood pressure; VEGF A: vascular endothelial growth factor A; WC: waist circumference.

It is noteworthy that in relation to biochemical parameters in group S, except for the correlation with leptin (see Table 7), body mass only correlated with serum levels at completion for IL1-alpha and this was only a moderate correlation (for baseline body mass R = 0.31; for completion body mass R = 0.35). In group S, there were no correlations between BMI and biochemical parameters (except some correlations with leptin, see Table 7), neither at baseline, nor at study completion. In group K, body mass did not correlate with biochemical parameters and %FTC and FTC correlated only with serum leptin levels (see Table 8). This shows that, despite differences in body mass between study onset and completion in group S and between groups S and K, body mass did not have a significant influence on the study results.

Linear regression analysis (y = β_1_x + β_0_) revealed, that there was a significant linear relationship between serum levels of leptin (x) and VEGF A (y) in group S before (VEGF A = 33.21leptin + 40.89) and after (VEGF A = 27.89leptin + 92.52) the intervention. No such relationship was found in group K. Linear regression analysis results are shown in Table 9.

**Table 9:** The linear regression model (y = β_1_x + β_0_) of the relationship between leptin (x) and VEGF A (y) serum levels.

Regression model parameter	Group S before the intervention		Group S after the intervention		Group K	
β	β_0_ = 40.89	β_1_ = 33.21	β_0_ = 92.52	β_1_ = 27.89	β_0_ = 127.98	β_1_ = − 1.52
SE	20.18	5.49	20.86	5.5	18.62	3.24
p	** < 0.0001**		**< 0.0001**		0.6419	
R	0.66		0.59		0.07	
R^2^	0.44		0.35		0.005	

R: correlation coefficient; R^2^: R squared; SE: standard error.

Significant values are given in bold.

### STEMI and NSTEMI comparison of rehabilitated patients

ACS usually has a form of ST-segment elevation myocardial infarction (STEMI) or non-ST- segment elevation myocardial infarction (NSTEMI); these two forms of ACS differ significantly in the range of pathophysiology, treatment and mortality^41^. In group S, there were 33 subjects who underwent ACS in the form of STEMI and 16 subjects who underwent ACS in the form of NSTEMI. According to this, group S was divided into STEMI and NSTEMI subgroups for further comparison. There was no significant difference in the patient sex distribution between the STEMI and NSTEMI subgroups (STEMI subgroup: women 10, men 23; NSTEMI subgroup: women 6, men 10; Fisher’s p-value = 0.5275).

In the range of anthropometric, body composition, blood pressure and heart rate results, STEMI patients had a higher DBP compared to NSTEMI patients at the completion of the intervention. There were no further inter-subgroup differences in group S. NSTEMI patients had a lower FFM, MM and BMR at completion compared to study onset; there were no such differences in STEMI patients. Conversely, STEMI patients were characterised by lower SBP and DBP after the intervention compared to baseline and there were no such differences in NSTEMI patients. The anthropometric, body composition, blood pressure and heart rate results of STEMI and NSTEMI patients who underwent cardiac rehabilitation are presented in Table 10.

**Table 10:** Comparison of anthropometric, body composition analysis, resting blood pressure and resting heart rate results between STEMI and NSTEMI patients in subjects from group S.

Parameter	STEMI (n = 33) /NSTEMI (n = 16)	Before intervention	After intervention	p-value
		Median [Q1; Q3]	Median [Q1; Q3]	
Body mass [kg]	STEMI	78.20 [67.50;86.10]	77.65 [68.60;86.35]	0.0903*
	NSTEMI	77.40 [64.80;83.40]	78.60 [64.40;83.40]	0.2094*
	p-value	0.8604^#^	0.6087^#^	
BMI [kg/m^2^]	STEMI	26.40 [23.29;30.30]	26.97 [23.35;30.95]	0.0511*
	NSTEMI	26.24 [23.90;32.40]	26.57 [23.10;27.90]	0.1078*
	p-value	0.7084^#^	0.7716^#^	
HC [cm]	STEMI	102.00 [97.00;109.50]	100.50 [97.00;109.00]	0.4077*
	NSTEMI	100.50 [97.00;111.00]	100.00 [96.00;112.00]	0.7598*
	p-value	0.9694^#^	1.0000^#^	
WC [cm]	STEMI	97.50 [91.00;108.00]	99.50 [91.50;107.25]	0.8314*
	NSTEMI	97.50 [95.00;104.00]	98.00 [87.00;104.00]	0.1415*
	p-value	0.8519^#^	0.5279^#^	
%FTC [%]	STEMI	31.40 [27.40;37.30]	30.65 [25.40;37.00]	0.6541*
	NSTEMI	27.10 [22.50;42.30]	32.50 [25.50;42.20]	0.0593*
	p-value	0.5872^#^	0.8362^#^	
FTC [kg]	STEMI	23.30 [21.20;30.95]	23.35 [19.70;31.50]	0.7228*
	NSTEMI	20.10 [17.50;35.20]	22.30 [19.60;31.90]	0.0926*
	p-value	0.3897^#^	0.8362^#^	
FFM [kg]	STEMI	52.20 [45.40;61.50]	52.90 [42.80;61.10]	0.2813*
	NSTEMI	56.40 [48.20;61.90]	53.80 [43.80;56.30]	0.0051*
	p-value	0.8201^#^	0.6382^#^	
MM [kg]	STEMI	29.15 [24.60;33.70]	29.25 [23.00;33.50]	0.1506*
	NSTEMI	30.80 [25.90;34.40]	29.50 [23.60;31.00]	0.0050*
	p-value	0.7662^#^	0.6382^#^	
BMR [kcal]	STEMI	1497.50 [1350.00;1698.50]	1512.00 [1294.00;1691.00]	0.2443*
	NSTEMI	1589.00 [1410.00;1706.00]	1533.00 [1316.00;1586.00]	0.0051*
	p-value	0.8201^#^	0.6115^#^	
SBP [mmHg]	STEMI	120.00 [110.00;135.00]	115.00 [101.00;130.00]	0.0190*
	NSTEMI	130.00 [115.00;140.00]	114.00 [93.00;141.00]	0.0843*
	p-value	0.3206^#^	0.9715^#^	
DBP [mmHg]	STEMI	76.00 [66.00;86.00]	72.00 [65.00;81.00]	0.0306*
	NSTEMI	70.00 [59.00;75.00]	64.00 [58.00;75.00]	0.0844*
	p-value	0.1315^#^	**0.0489**^#^	
HR [bpm]	STEMI	67.00 [64.00;76.00]	65.50 [60.00;72.00]	0.1619*
	NSTEMI	62.00 [54.00;68.00]	63.00 [56.00;72.00]	0.4603*
	p-value	0.0581^#^	0.4105^#^	

Data are presented as median [Q1; Q3]. %FTC: percentage fat tissue content; BMI: body mass index; BMR: basal metabolic rate; bpm: beats per minute; DBP: resting diastolic blood pressure; FFM: fat-free mass; FTC: mass fat tissue content; HC: hip circumference; HR: resting heart rate; MM: muscle mass; NSTEMI: non-ST-segment elevation myocardial infarction; Q1: first quartile; Q3: third quartile; SBP: resting systolic blood pressure; STEMI: ST-segment elevation myocardial infarction; WC: waist circumference.

*Wilcoxon test; ^#^Mann–Whitney test.

Significant values are given in bold.

Considering biochemical outcomes, both in the STEMI and NSTEMI subgroups, serum VEGF A levels were higher at study completion compared to baseline. In STEMI patients, serum HIF-1A and VEGF R2 concentrations were lower at the end of the intervention compared to study onset. There were no other significant intra- and inter-subgroup differences for the STEMI and NSTEMI subgroups in the range of biochemical outcomes. The serum biochemical results of STEMI and NSTEMI patients who underwent cardiac rehabilitation are presented in Table 11.

**Table 11:** Comparison of serum concentration of biochemical parameters between STEMI and NSTEMI patients in subjects from group S.

Parameter	STEMI (n = 33)/NSTEMI (n = 16)	Before intervention	After intervention	p-value
		Median [Q1; Q3]	Median [Q1; Q3]	
VEGF A [pg/ml]	STEMI	72.55 [50.59; 141.99]	117.77 [75.14; 147.24]	0.0034*
	NSTEMI	57.76 [47.46; 173.32]	126.37 [90.88; 182.88]	0.0076*
	p-value	0.8777^#^	0.4022^#^	
Leptin [ng/ml]	STEMI	1.65 [1.20; 2.43]	1.00 [0.40; 2.62]	0.0961*
	NSTEMI	1.86 [1.09; 4.62]	1.45 [0.55; 2.92]	0.0609*
	p-value	0.4275^#^	0.5231^#^	
HIF-1A [pg/ml]	STEMI	64.16 [25.76; 107.74]	24.14 [14.98; 83.34]	0.0299*
	NSTEMI	50.51 [28.24; 317.00]	22.06 [11.36; 733.18]	0.3066*
	p-value	0.8604^#^	0.9825^#^	
NfkB [ng/ml]	STEMI	0.34 [0.27; 0.48]	0.26 [0.23; 0.46]	0.2388*
	NSTEMI	0.36 [0.29; 0.48]	0.26 [0.22; 0.41]	0.2585*
	p-value	0.8632^#^	0.7915^#^	
Notch 1 [pg/ml]	STEMI	713.63 [643.14; 937.36]	726.76 [617.30; 1085.20]	0.8793*
	NSTEMI	718.53 [605.86; 795.57]	738.12 [620.22; 973.41]	0.1118*
	p-value	0.4947^#^	0.9825^#^	
IL1-alpha [pg/ml]	STEMI	30.87 [22.42; 37.13]	34.49 [26.30; 38.12]	0.1404*
	NSTEMI	35.47 [26.62; 112.66]	28.28 [21.10; 61.02]	0.2114*
	p-value	0.0644^#^	0.3898^#^	
VEGF R2 [ng/ml]	STEMI	0.23 [0.14; 0.30]	0.17 [0.15; 0.21]	0.0136*
	NSTEMI	0.18 [0.13; 0.22]	0.17 [0.13; 0.23]	0.6247*
	p-value	0.1371^#^	0.6599^#^	

Data are presented as median [Q1; Q3]. HIF-1A: hypoxia-inducible factor 1 alpha; IL1-alpha: interleukin 1 alpha; NfkB: nuclear factor kappa-light-chain-enhancer of activated B cells; Notch 1: Notch protein 1; NSTEMI: non-ST-segment elevation myocardial infarction; Q1: first quartile; Q3: third quartile; STEMI: ST-segment elevation myocardial infarction; VEGF A: vascular endothelial growth factor A; VEGF R2: vascular endothelial growth factor receptor 2.

*Wilcoxon test; ^#^Mann–Whitney test.

Significant values are given in bold.

## Discussion

To the best of our knowledge, ours is the first study to show that CR leads to a decrease in serum leptin levels in patients after ACS. Moreover, our outcomes revealed that ACS may disrupt leptin-dependent VEGF A serum level regulation and CR manages to at least partially restore this regulation. These two outcomes constitute the key findings and the novelty of the study.

### Leptin and VEGF A

Increased serum leptin levels are significantly associated with CAD^42^. Due to the increasing amount of scientific evidence, leptin began to be seen not as a marker but as a causative agent of CAD, mediating atherosclerotic processes^43^ independent from traditional cardiovascular risk factors and from body mass status^13,14^. Serum leptin levels positively correlate with the severity of CAD being higher in patients with stable angina compared to controls and the highest in subjects with unstable angina^44^. Higher serum leptin concentrations were associated with arterial stiffness and an increasing quantity of stenotic coronary arteries in CAD patients^45^. Finally, acute MI also elevates blood leptin concentration significantly^15^. The deregulation of leptin metabolism exerts a detrimental effect on the heart, affecting cardiac remodelling, contractile function and cardiac metabolism^46^. Also, leptin enhances platelet activation in patients with CAD^47^.

Our study revealed that short-term CR leads to a decrease in serum leptin levels in patients after ACS. Such a result constitutes a novelty, as studies performed so far in this topic have shown that CR only prevents an increase in blood leptin levels in this group of subjects^24^. Interestingly, the outcome of the current study was registered despite the increase in body mass and BMI in the training group. Of note, there were no differences in HC, WC, %FTC and FTC between study completion and baseline in group S. Also, there are no correlations between the change in leptin serum levels and body composition parameters in this group. Thus, decrease serum leptin levels in patients after ACS resulting from short-term CR are not associated with alterations in fat tissue content. Interestingly, both SBP and DBP, well-known factors of cardiovascular risk^48^, were lower after CR compared to study onset in group S. However, serum leptin level decreases in this group were negatively correlated with SBP and DBP decreases. This outcome revealed that the favourable effect of a leptin blood concentration reduction on the cardiovascular system resulting from CR in patients after ACS is not mediated through blood pressure decreases. Taking all of this together, our results suggest that the beneficial effect of short-term CR on cardiovascular system and cardiovascular risk in patients after ACS is mediated in some range by the decrease in serum leptin levels; also, this effect seems to be independent of fat tissue content and blood pressure alterations. It is worth mentioning that the leptin serum levels were significantly higher in group K compared to the baseline leptin levels in group S. As in group K the time range between ACS and inclusion into the study was longer than in group S this result shows, that in patients after ACS left without CR serum leptin level increases, which can exert detrimental effect on the whole cardiovascular system^43–45^.

Patients suffering from CAD and ACS due to endothelial damage are characterised by elevated VEGF A serum levels^29^, which is the basis of the proangiogenic cardiovascular system repair mechanism observed in subjects after ACS^28^. In our study, we have shown that serum VEGF A levels were higher after the rehabilitation compared to baseline in cardiac rehabilitated patients. Our results confirmed previous observations that CR leads to blood VEGF A concentration increases in CAD patients^30^, which may be associated with a proangiogenic response and amelioration of endothelial function as an effect of CR^30^.

So far, little is known about mutual dependence between leptin and VEGF in patients with CAD. Takahashi et al. observed that both leptin and VEGF serum levels are higher in CAD patients compared to in non-CAD subjects, which suggests their involvement in the development of coronary atherosclerosis. The authors suggest that leptin regulates the expression of VEGF influencing the pathogenesis of coronary atherosclerosis^49^. Recent animal and in vitro studies by Abd Alkhaleq et al. proved that leptin increases the level of VEGF and HIF-1A messenger ribonucleic acid (mRNA) in the heart^50^. However, no human studies investigating the link between leptin and VEGF in humans, especially patients with CAD after ACS, have been performed. In our study, we have shown a positive correlation between leptin and VEGF A serum levels at study completion in group S. Such dependence has not been registered for baseline leptin. Also, there was no correlation between serum leptin and VEGF A levels in group K. Linear regression analysis has shown relationship between serum levels of leptin and VEGF A in group S both before and after the CR and no such relationship in group K. This result only confirms, that the relation between these two substances needs scientific investigation. Our observations allow us to presume that ACS may disrupt mutual regulation between leptin and VEGF A and CR is able to restore it. Based on previous in vitro and animal studies^32–34,50,51^, we hypothesised that HIF-1A, NfkB and NILCO are the molecules and mechanisms which are substantially involved in the influence of leptin on VEGF A in patients after ACS undergoing CR.

### HIF-1A and NfkB

The up-regulation of VEGF expression by leptin involves the activation of HIF-1A and NfkB by leptin canonical (phosphatidylinositol-3 kinase/protein kinase B [PI-3K/AKT1] and mitogen-activated protein kinase/extracellular signal-regulated kinases 1 [MAPK/ERK1]) and non-canonical (c-Jun N-terminal kinase [JNK], p38 MAP kinase and to a less extent, protein kinase C [PKC]) signalling pathways. Also, leptin, through canonical signalling pathways (MAPK, PI-3K and Janus kinase 2/signal transducer and activator of transcription 3 [JAK2/STAT3]) induces the transcription factor SP1, which also plays a role in VEGF gene regulation. PKC activation is transferred to MAPK/ERK and p38 kinases, which further activates HIF-1A and NfkB and enhances the expression of VEGF^32^.

In our study, we have shown that serum levels of HIF-1A after CR were lower compared to baseline in group S. In the case of NfkB, there was no such a difference. In group S before CR, serum levels of HIF-1A and NfkB did not differ from those of group K. However, in group K, serum levels of both HIF-1A and NfkB were higher compared to group S at completion. This shows that CR decreases the serum HIF-1A levels in patients after ACS. Also, these results seem to indicate that even short-term CR reduces the serum level of HIF-1A and NfkB below the level observed in subjects after ACS without CR.

Our study showed no correlation between serum levels of leptin with levels of both HIF-1A and NfkB in groups S and K. This means that ACS may dysregulate the canonical and non-canonical leptin signalling pathways and CR did not manage to restore it. Also, in both group K and group S before CR, serum concentrations of VEGF A did not correlate with levels of HIF-1A and NfkB. This allows us to presume that ACS disrupts the influence of HIF-1A and NfkB on VEGF A. In group K, a strong positive correlation between serum levels of HIF-1A and NfkB confirms the close relationship of these two substances in regulatory pathways. In group S, CR restored the correlation of VEGF A blood level with HIF-1A and NfkB concentrations and the increase in VEGF A level was the greater the greater the baseline HIF-1A level was. Taking into consideration that leptin and VEGF A serum levels in group S were correlated after CR, it can be presumed that CR restored the influence of leptin on VEGF A synthesis regulated by HIF-1A and NfkB, but with the use of signalling pathways different from the canonical and non-canonical leptin signalling pathways.

### NILCO

It has been recently shown that leptin up-regulates VEGF R2 in endothelial cells. Also, leptin increases VEGF/VEGF R2 expression via the up-regulation of Notch and IL1, forming the Notch, IL1 and leptin cross-talk outcome (NILCO) pathway. Moreover, leptin induces the expression of Notch1-4/Jagged-1/delta-like canonical Notch ligand (Dll) 4, which plays an important role in Notch signalling pathway^33,51,52^. Furthermore, some studies indicated, that IL1 activates Notch signalling through NfkB activity^53^ and leptin up-regulates IL1 via an NfkB-dependent mechanism^32^. These effects are closely related to the JAK2/STAT3 signalling pathway^33^ inducing VEGF synthesis via the transcription factor SP1^32^. All of these data indicating the complex influence of leptin on VEGF were obtained from in vitro and animal studies. Our trial is the first human study attempting to register and at least partially clarify the dependence between leptin and VEGF A in humans, namely in patients after ACS undergoing CR.

In our study, serum levels of VEGF R2 in group S were lower after CR compared to baseline. Despite the lack of a direct correlation between leptin and VEGF R2 serum levels, we can presume that the decrease in serum leptin levels resulting from CR is associated with the decrease in serum VEGF R2 levels in patients after ACS. We can therefore hypothesise that this decrease in receptor levels constitutes a kind of buffer mechanism counteracting the effects of serum VEGF A level elevation in these subjects. There were no further intra- or inter-group differences in the serum levels of VEGF R2, Notch 1 and IL1-alpha.

Correlations registered in our trial at least partially confirmed the participation of NILCO in the regulation of VEGF A serum levels mediated by HIF-1A and NfkB. In group S the higher were baseline serum levels of IL1-alpha and Notch 1 the lower was the decrease in serum level of HIF-1A; the lower was the decrease in serum level of leptin the higher was completion serum level of Notch 1; the higher was baseline serum concentration of IL1-alpha the higher was serum level increase of VEGF A. In group S after CR, we registered a positive correlation between serum levels of HIF-1A and IL1-alpha; the same correlation was also shown in group K. However, in group S, but not in group K, and only after CR, we showed a positive correlation between serum levels of NfkB and Notch 1. We can presume that it reflects the previously described activation of Notch signalling through NfkB activity^53^, which is restored in patients after ACS by CR.

Taking all of these data together, it is possible to hypothesise that the NILCO pathway, interacting with HIF-1A and especially with NfkB, predominates over leptin canonic and non-canonic pathways in the leptin influence on VEGF A, in patients after ACS who underwent CR.

### STEMI vs NSTEMI

After the study intervention, DBP was lower in the NSTEMI subgroup compared to the STEMI subgroup. However, in the STEMI subgroup, both SBP and DBP were lower after the study intervention compared to baseline, whereas there were no such differences in the NSTEMI subgroup. Thus, as BP is a well-known cardiovascular risk marker^54^, we can presume that STEMI patients benefited more from CR than NSTEMI subjects.

In both the STEMI and NSTEMI subgroups, serum VEGF A levels were significantly higher after the intervention compared to baseline and serum leptin levels tended to be lower after CR compared to study onset (however, there was no statistical significance). This subgroup analysis confirms changes observed in the whole of group S. Only in the STEMI subgroup were HIF-1A and VEGF R2 serum levels lower at study completion compared baseline. This seems to indicate that the influence of cardiac rehabilitation on leptin and VEGF A cross-talk is more strongly pronounced in STEMI patients than in NSTEMI cases. However, the lack of statistical significance in the NSTEMI subgroup in the range of HIF-1A and VEGF R2 serum level results might be a consequence of the relatively low number of subjects in this subgroup.

### Study strong points

The strongest point of the study results from its novelty; to the best of our knowledge, ours is the first trial investigating leptin and VEGF A cross-talk in humans after ACS, especially in those undergoing cardiac rehabilitation. Moreover, in the study we made attempts to investigate not only HIF-1A and NfkB-dependent leptin influence on VEGF A, but also the NILCO pathway and the effect of leptin on VEGF R2 comparing the human results with a range of previous in vitro studies. The second strongest point of the study is the relatively high number of subjects included, both in the intervention and control groups. Our trial may open up a new perception of how cardiac rehabilitation influences the function of the cardiovascular system in subjects after ACS and in the future could help to improve cardiac rehabilitation programmes to increase their beneficial effects in this group of patients.

### Study limitations

The greatest limitation of the study is the relatively short duration of the CR programme. However, a previous meta-analysis showed a significant influence of a short-term 2-week physical exercise programme on leptin blood concentrations independent of age and sex^21^. Despite the study intervention lasting for only 2 weeks, our study managed to reveal a range of significant results allowing a consistent analysis and interpretation of the data. A range of biochemical parameters which serum concentration was determined in the study with the use of ELISA are intracellular transcription factors—in this range HIF-1A and NfkB or membrane proteins—Notch 1 and VEGF R2. In case of these parameters immunohistochemistry tissue analysis or Western Blot cellular analysis would be more adequate methods of content determination than ELISA. However, ELISA allowed us to perform the study in a quite large population of patients in a specific clinical state- namely after ACS. Also, we managed to analyze a broad range of factors involved in leptin and VEGF cross-talk in human study. In the study signal transduction analysis, such as axis of PI-3K/AKT1, MAPK/ERK1, JNK, p38 MAP kinase or PKC; and VEGF receptor, HIF-1A, NfkB, and Notch 1 analysis on peripheral blood mononuclear cells, has not been performed. It would be valuable to perform such analysis in future scientific investigations in this topic.

## Conclusions

A 2-week cardiac rehabilitation programme in patients after acute coronary syndrome increases the serum levels of VEGF A and decreases blood concentrations of leptin. ACS may disrupt leptin and VEGF A cross-talk, but a 2-week cardiac rehabilitation programme manages to restore it. In patients after ACS, 2 weeks of cardiac rehabilitation seems to result in the predomination of the NILCO pathway interacting with HIF-1A and especially with NfkB over the canonical and non-canonical leptin pathways in the influence of leptin on VEGF A. STEMI patients might have benefited more from a 2 week cardiac rehabilitation programme compared to NSTEMI subjects, but this issue requires further scientific investigation.

## Data Availability

The datasets used and analysed during the current study are available from the corresponding author on reasonable request.

## Abbreviations

%FTC: Percentage fat tissue content
%GR: Percent grade
6MWT: 6 minutes walk test
ACC/AHA: American College of Cardiology/American Heart Association
ACS: Acute coronary syndrome
BMI: Body mass index
BMR: Basal metabolic rate
BP: Blood pressure
BPmax: Maximum blood pressure
CABG: Coronary artery bypass grafting
CAD: Coronary artery disease
CHF: Congestive heart failure
CPX: Cardiac stress test
CR: Cardiac rehabilitation
CV: Cardiovascular
DBP: Diastolic blood pressure
DES: Drug eluting stent
Dll: Notch1-4/Jagged-1/delta-like canonical Notch ligand
ECG: Electrocardiographic, electrocardiogram
ELISA: Enzyme-linked immunosorbent assay
ESC: European Society of Cardiology
FFM: Fat-free mass
FTC: Mass fat tissue content
HC: Hip circumference
HIF-1A: Hypoxia-inducible factor 1 alpha
HR: Heart rate
HRmax: Maximum heart rate
HRR: Heart rate reserve
HRrest: Resting heart rate
IHD: Ischemic heart disease
IL1: Interleukin 1
IL1-alpha: Interleukin 1-alpha
JAK2/STAT3: Janus kinase 2/signal transducer and activator of transcription 3
JNK: C-Jun N-terminal kinase
MACE: Major adverse cardiac events
MAPK/ERK1: Mitogen-activated protein kinase/extracellular signal-regulated kinases 1
MET: Metabolic equivalent of task
MI: Myocardial infarction
MM: Muscle mass
mph: Miles per hour
mRNA: Messenger ribonucleic acid
NfkB: Nuclear factor kappa-light-chain-enhancer of activated B cells
NILCO: Notch, interleukin 1 and leptin cross-talk outcome
Notch: Notch protein
NSTEMI: Non-ST-segment elevation myocardial infarction
PCI: Percutaneous coronary intervention
PCS: Polish Cardiac Society
PI-3K/AKT1: Phosphatidylinositol-3 kinase/protein kinase B
PKC: Protein kinase C
POBA: Plain old balloon angioplasty
RR: Relative risk
SBP: Systolic blood pressure
SE: Standard error
SO_2_: Blood oxygen saturation
STEMI: ST-segment elevation myocardial infarction
VEGF: Vascular endothelial growth factor
VEGF A: Vascular endothelial growth factor A
VEGF R2: Vascular endothelial growth factor receptor 2
WC: Waist circumference
